# Fente mandibulaire médiane chez l’adulte: à propos d’un cas et revue de la littérature

**DOI:** 10.11604/pamj.2021.38.257.26392

**Published:** 2021-03-11

**Authors:** Bouchra Dani, Zahra Sayad, Malik Boulaadas

**Affiliations:** 1 Service de Chirurgie Maxillo-faciale et Stomatologie, Centre Hospitalier Universitaire Ibn Sina, Rabat, Maroc

**Keywords:** Fente mandibulaire, fente sternale, fente n^o^30 de Tessier, à propos d’un cas, Mandibular cleft, sternal cleft, Tessier's cleft No. 30, case report

## Abstract

Les fentes oro-faciales sont des malformations congénitales fréquentes. La classification la plus utilisée est celle de Tessier qui comprend 30 variantes. Dont les fentes mandibulaires médianes (fente n°30 de Tessier) isolées ou accompagnées d´une fente de la lèvre inférieure, de la langue ou rarement du sternum. Elles sont très rares, moins de 70 cas (toutes formes confondues) ont été décrits dans la littérature. Nous rapportons un cas exceptionnel d´une fente mandibulo-sternale et nous faisons une revue de la littérature.

## Abstract (en)

Orofacial clefts are common congenital malformations. Tessier's classification system is the most widely used; it consists of 30 variants including median mandibular clefts (Tessier's cleft No. 30). They can be isolated or associated with cleft of the lower lip, tongue or more rarely, of the sternum. They are very rare, less than 70 cases (all types taken together) have been reported in the literature. We here report an exceptional case of mandibular and sternal cleft with literature review.

## Introduction

Les fentes mandibulaires appartiennent au groupe des malformations oro-faciales d´origine congénitale. Elles sont très rares, moins de 70 cas ont été rapportés dans la littérature [1]. La classification la plus utilisée est celle de Tessier publiée en 1976 [2], dans laquelle la fente mandibulaire est classée « Tessier 30 ». Les fentes classées n°30 de Tessier regroupent, les fentes mandibulaires médianes isolées ou accompagnées d´une atteinte de la lèvre inférieure, de la langue et/ou d´autres tissus mous jusqu´au manubrium sternal [2]. Elles sont très rares, leur incidence étant estimée entre 1,4 et 4,9 pour 100 000 naissances vivantes [3,4]. Nous rapportons un cas exceptionnel de fente mandibulaire et sternale proprement dite avec une revue de la littérature.

## Patient et observation

**Figure 1:**
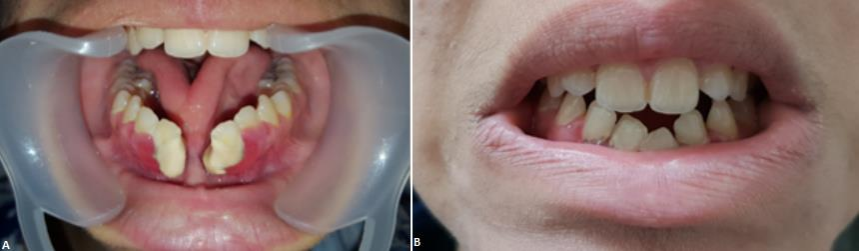
photos de la patiente montrant; A) aspect de 2 hémi-mandibule et la continuité entre le frein lingual et du vestibule inférieur formant une ankyloglossie; B) aspect de l´articulé 2 ans après la chirurgie

**Figure 2:**
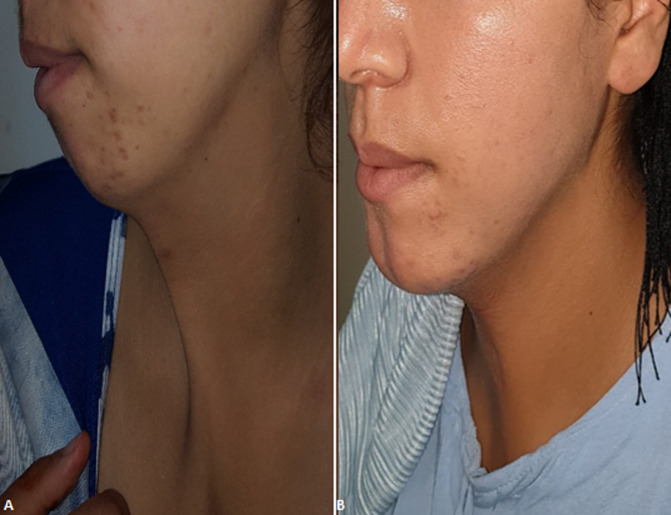
photos de la patiente montrant: A) ride cervicale allant du menton jusqu'à la fourchette sternale; B) aspect du cou 2 ans après la chirurgie

**Figure 3:**
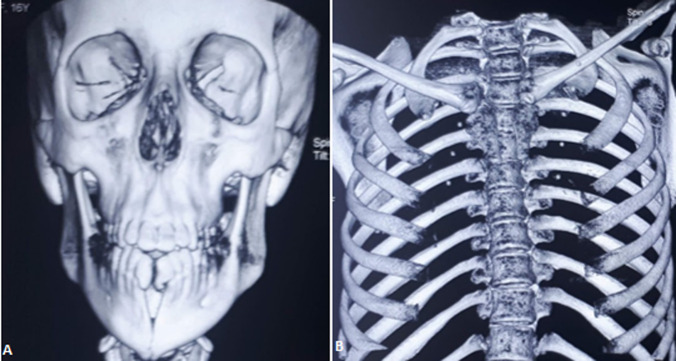
tomodensitométrie du massif facial et thoracique montrant: A) fente mandibulaire; B) fente sternale

**Figure 4:**
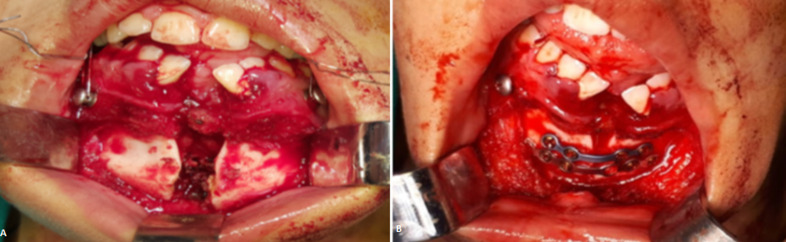
images per opératoires: A) frénoplastie de l´ankyloglossie et décollement sous périosté de la muqueuse; B) ostéosynthèse par 2 mini-plaques 4 trous décalés

**Figure 5:**
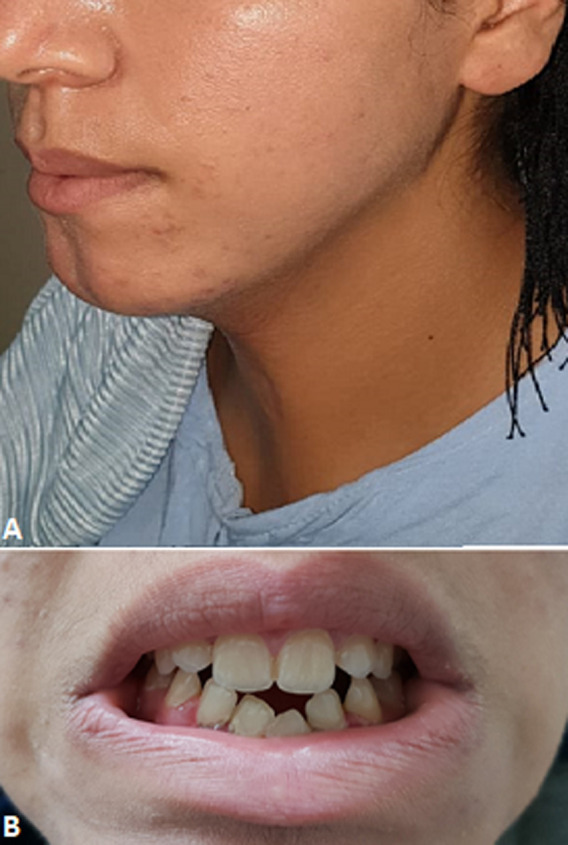
photos de la patiente 2 ans après la chirurgie; A) aspect du cou; B) aspect de l´articulé

Nous rapportons le cas d´une fille de 20 ans, sans antécédent pathologique médical ni chirurgical qui était prise en charge au sein de notre service pour une fente mandibulaire congénitale. À l´examen clinique la mandibule donnait l´aspect de 2 hémi-mandibules, la lèvre inférieure était d´aspect normal. L´examen endobuccal retrouvait une continuité entre le frein lingual et du vestibule inférieur, formant ainsi une ankyloglossie (Figure 1). La patiente présentait aussi une bride cervicale allant du menton jusqu'à la fourchette sternale (Figure 2 A,B). Une tomodensitométrie du massif facial et thoracique avait mis en évidence une fente mandibulaire et sternale (Figure 3 A,B). Un bilan complet était réalisé à la recherche d´autres malformations associées revenait normal. La patiente a bénéficié d´une chirurgie de réduction pour sa fente mandibulaire. D´abord on a réalisé une frénoplastie pour l´ankyloglossie (Figure 4 A). Suivi d´une ostéosynthèse mandibulaire par 2 mini-plaques 4 trous décalés et 8 mini vis 7 mm, sans nécessité d´un greffon osseux (Figure 4 B). Une plastie en Z a été réalisée pour sa bride cervicale. L´évolution de notre patiente était favorable avec un recul de 2 ans (Figure 5 A). L´ostéosynthèse mandibulaire est en place et la région cervicale est devenue normale (Figure 5 B). On a proposé à notre patiente un traitement orthodontique pour l´alignement des dents.

## Discussion

Les fentes médianes mandibulaires et de la lèvre inférieure, classé Tessier 30, sont des anomalies congénitales rarement rapportées dans la littérature. Le 1^er^ cas a été décrit par COURONNE en 1819 [5]. Une revue de la littérature par Erdogan *et al*. en 1989 [6] a trouvé 48 patients présentant une fente médiane de la lèvre inférieure dont 37 cas associaient aussi la mandibule. Armstrong et Waterhouse [7] ont rapporté 67 cas de fentes médianes de la lèvre inférieure avec une fente mandibulaire associé. La dernière revue de la littérature faite par Benhamou en 2006 [1] a trouvé 70 cas de fentes classées n°30 de Tessier. Par la suite, juste quelques cas ont été publiés, mais aucun cas de fente mandibulaire isolée chez un adulte n´a été rapporté. La fente n°30 de Tessier est une anomalie embryologique, il s´agirait d´une anomalie de fusion du premier arc branchial, ce qui explique que la fente peut intéresser tous les organes provenant de cet arc. La complexité de la malformation est donc variable selon le moment de l´anomalie embryologique [1].

Plusieurs hypothèses concernant l´étiopathogénie de cette malformation ont été décrites dans la littérature. La plupart des auteurs considèrent que cette anomalie résulte d´un échec de fusion de la première paire d´arcs branchiaux, ou c´est un échec de pénétration mésodermique dans la ligne médiane de la partie mandibulaire du premier arc branchial [3]. Morton et Jordan [8] estiment que la deuxième théorie pourrait expliquer l´absence de l´os hyoïde, de cartilage thyroïdien, des muscles hyoïdiens et du manubrium dans les variétés les plus graves. En 1996, Oostrom *et al*. [9] ont proposé qu'il n'y ait qu'un seul arc branchial pendant les premières périodes embryonnaires (7^e^ semaine) dans lequel 2 ébauches mandibulaires se développent avec un sillon au centre. Ainsi, les malformations mineures surviendraient tardivement au cours de la vie embryonnaire, et inversement, les formes sévères seraient dues à un défaut de migration des 2 ébauches mandibulaires au tout début de la vie embryonnaire [10]. La revue de la littérature nous a permis de retrouver des formes dont la sévérité est très variable ainsi que la présentation clinique.

Dans les formes mineures, seule la lèvre inférieure peut être atteinte mais généralement, la fente s'étend jusqu'à la symphyse mandibulaire, la langue peut être bifide et fixée à la fente alvéolaire et parfois on trouve une ankyloglossie [2]. Dans les formes sévères, l'épiglotte ou l´os hyoïde peuvent être absente et le cartilage thyroïdien peut être sous-développé [3]. Parfois, il peut y avoir un cordon cervical médian [11] comme dans notre cas. Les muscles hyoïdiens peuvent être atrophiques et représentés par un tissu fibreux provoquant un bombement du cou au cours de l'effort [10]. Les clavicules peuvent être largement espacés et le manubrium sternal peut être bifide (comme dans notre cas) ou absent [8]. En 2001 Seyhan *et al*. [12] ont rapporté un cas de fente sternale associé à une cardiopathie, aussi en 2018 Ahmed Ali [11] a rapporté un cas de fente mandibulaire associé à une malformation cardiaque. À la lumière de ces données un bilan complet à la recherche d'autres malformations congénitales associées chez notre patiente a été réalisé, revenu complètement normal. Tous les cas rapportés dans la littérature intéressent des nourrissons, un seul cas de diagnostic tardif à l´âge de 17 ans a été décrit, présentant une simple fissure médiane de la lèvre inférieure sans atteinte osseuse ou de la langue [12]. Notre cas est une forme exceptionnelle d´une fente n°30 de Tessier chez un adulte de 20 ans. Intéressant uniquement la mandibule et le sternum avec une ankyloglossie ainsi qu´une bride cervicale médiane.

La rareté et la variabilité de la gravité de cette affection sont responsables de l'absence de consensus thérapeutique nettement codifié et du moment idéal pour intervenir chirurgicalement. Le problème se pose surtout dans les fentes mandibulaires chez les nourrissons. La question qui se pose, à quel âge doit-on aborder la mandibule pour éviter une altération des germes dentaires? Ce problème ne se posait pas chez notre patiente puisqu´elle avait sa denture définitive. Mais dans la littérature on trouve différentes attitudes. Par exemple Millard *et al*. [13] ont opté pour une correction initiale de la fente médiane de la lèvre inférieure à 6 mois, suivi d´une ostéosynthèse mandibulaire à 8 ans, puisqu´ils considèrent qu´une fermeture précoce du défaut mandibulaire peut condamner les germes dentaires. Sherman et Goulian [14] ont traité avec succès une fente mandibulaire à l'âge de 20 mois. Aussi, Oostrom *et al*. [9] confirme qu'une ostéosynthèse prudente de la base de la mandibule n´aura aucun retentissement sur les germes dentaires et il en résulte une meilleure occlusion dentaire. Quant aux pertes de substances muqueuses, elles sont réparées soit par des sutures simples voire des greffes muqueuses ou cutanées. Pour les ankyloglossies, on réalise des frénotomies ou des frénoplasties.

## Conclusion

Dans ce travail nous avons rapporté un cas exceptionnel d´une fente mandibulaire isolée chez un adulte. Les fentes n°30 de Tessier sont des anomalies congénitales très rares, leur prise en charge doit être précoce et les indications chirurgicales doivent être discutées au cas par cas.
